# Impact of a clinical decision protocol on survival and neurological outcome following extracorporeal cardiopulmonary resuscitation

**DOI:** 10.1186/s40560-026-00874-7

**Published:** 2026-03-16

**Authors:** Zied Ltaief, Jean Bonnemain, Filip Dulguerov, Anna Nowacka, Lars Niclauss, Marco Rusca, Nawfel Ben-Hamouda, Pierre-Nicolas Carron, Valentina Rancati, Patrick Yerly, Baudouin Bourlond, Matthias Kirsch, Lucas Liaudet

**Affiliations:** 1 The Service of Adult Intensive Care Medicine, Lausanne University Hospital, Lausanne, 1010 Switzerland; 2 The Service of Cardiac Surgery, Lausanne University Hospital, Lausanne, 1010 Switzerland; 3 The Emergency Department, Lausanne University Hospital, Lausanne, 1010 Switzerland; 4 The Service of Anesthesiology, Lausanne University Hospital, Lausanne, 1010 Switzerland; 5 The Service of Cardiology, Lausanne University Hospital, Lausanne, 1010 Switzerland

**Keywords:** Extracorporeal cardiopulmonary resuscitation (ECPR), Veno-arterial membrane oxygenation (VA-ECMO), Cardiac arrest, Clinical protocol

## Abstract

**Background:**

Extracorporeal cardiopulmonary resuscitation (ECPR) can improve survival in patients with refractory cardiac arrest (CA). However, defining optimal selection criteria for ECPR remains a major challenge.

**Methods:**

We retrospectively analyzed all ECPR treatments for refractory in-hospital CA (IHCA) and out-of-hospital CA (OHCA) in adult patients from January 1, 2010 through December 31, 2024 at our tertiary 35-bed Intensive Care Unit. Before July 2017 (Period 1), ECPR was implemented at physician discretion. From July 2017 (Period 2), a dedicated protocol recommended physicians to implement ECPR based on four criteria: age < 70 years, shockable rhythm, no-flow duration < 5 min, and total low-flow duration < 80 min. The primary outcome was hospital mortality. The secondary outcome was good neurological outcome at 3 months, defined by a cerebral performance category (CPC) score of 1 or 2.

**Results:**

A total of 166 patients (45 in period 1, 121 in period 2), including 80 IHCAs and 86 OHCAs, were included. The proportion of patients fulfilling the 4 criteria was low yet significantly greater in period 2 than in period 1 (35.0 vs. 17.8%, *p* = 0.027). Hospital survival was improved in period 2 (26.5% vs. 8.9%, *p* = 0.015), whereas good neurological outcome was not (14.9 vs. 6.7%, *p* = 0.157). When evaluating the impact of the 4 criteria over the whole study period, patients with 4 criteria vs. those with < 4 criteria displayed marked improvements in survival (48.0 vs. 9.6%, *p* < 0.001) and good neurological outcome (30.0 vs. 5.2%, *p* < 0.001). In multivariable analysis, only the simultaneous presence of the 4 criteria was independently associated with a decreased risk of death (OR = 0.11, 95% CI 0.01–0.87, *p* = 0.037), whereas no single criterion alone was significantly predictive.

**Conclusion:**

Implementing a clinical ECPR protocol in our institutional practice improved meaningful survival in patients with refractory IHCA and OHCA fulfilling four predefined criteria including an age < 70 years, a shockable rhythm, a no-flow < 5 min, and a low-flow < 80 min.

**Supplementary Information:**

The online version contains supplementary material available at 10.1186/s40560-026-00874-7.

## Background

Refractory cardiac arrest (CA) despite high-quality conventional cardiopulmonary resuscitation (CPR) remains associated with extremely poor prognosis [1]. In this specific context, veno-arterial extracorporeal membrane oxygenation (VA-ECMO) can be initiated as extracorporeal cardiopulmonary resuscitation (ECPR) to rapidly restore systemic perfusion and provide a bridge to the diagnosis and treatment of potentially reversible causes of CA, such as coronary artery occlusion. ECPR may therefore limit organ damage and increase the probability of survival in selected patients with refractory out-of-hospital CA (OHCA) and in-hospital cardiac arrest (IHCA) [2]. The utilization of ECPR has increased substantially over the past decade [3]. While several meta-analyses and systematic reviews suggest that this strategy may improve survival and neurological outcomes in selected cases of both OHCA and IHCA [4–6], evidence supporting its widespread use in refractory CA remains insufficient. This uncertainty is highlighted by the conflicting results of three recent randomized trials on ECPR in OHCA scenario. In the ARREST trial, conducted in a highly controlled environment with strictly selected patients (shockable rhythms and minimal delays to ECPR), the technique showed significant efficacy, leading to a marked increase in survival [7]. However, this survival benefit was not observed in the Prague-OHCA trial, where inclusion criteria were broadened to patients with non-shockable rhythms [8]. Similarly, the INCEPTION trial, which included patients with shockable rhythms from non-metropolitan areas (resulting in longer delays to ECPR), also failed to demonstrate a survival benefit with ECPR [9]. The key finding from these three randomized-controlled trials is that selection criteria are paramount in determining the effectiveness of ECPR. The primary challenge for clinicians lies in accurately identifying patients who may truly benefit from ECMO before initiating an invasive procedure with high cost, complexity, and elevated risk of complications. In this paper, we present our center’s experience across two periods of ECPR use: the first without specific selection criteria and the second after implementing a protocol to guide clinicians selecting appropriate candidates for ECPR in refractory IHCA and OHCA.

## Methods

### Study setting and population

**Fig. 1:**
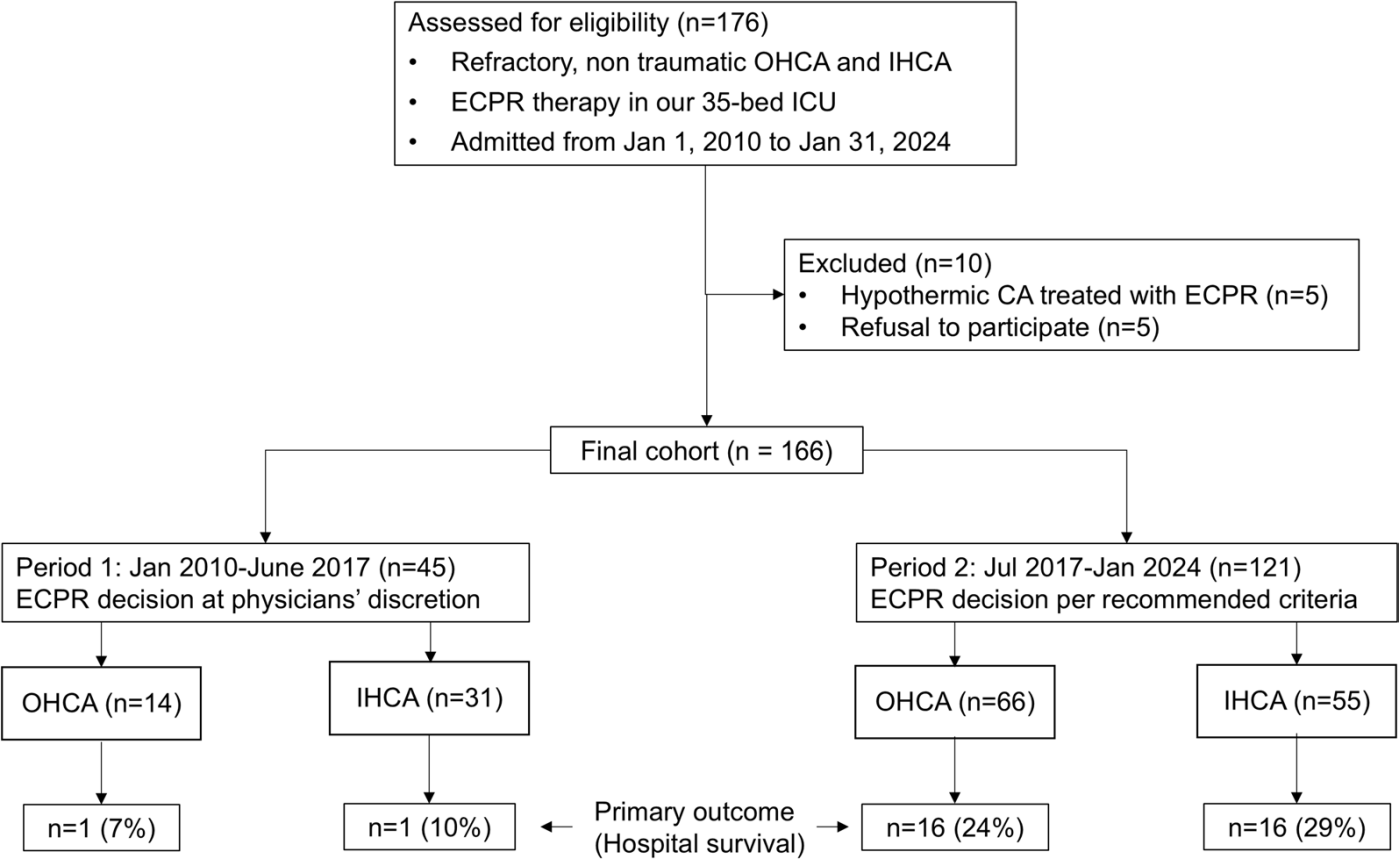
Study flowchart. *CA* Cardiac Arrest, *ECPR* Extracorporeal Cardio-Pulmonary Resuscitation, *IHCA* In-Hospital Cardiac Arrest, *ICU* Intensive Care Unit, *OHCA* Out-of-Hospital Cardiac Arrest

This study was approved by our ethical committee (Commission Cantonale d’Ethique de la Recherche sur l’Etre Humain/CER-VD-Nr: 2024-00887, ECPR-PRO Study), as a retrospective analysis of clinical variables, with the exclusion of patients who had specifically indicated their refusal to participate in any form of research. It represents a single-center, *before-and-after* observational analysis, evaluating the impact of implementing a clinical ECPR decision protocol within our institutional practice. The study conforms to the STROBE statement for the report of observational studies. Patients were included if they met the following criteria: refractory non-traumatic OHCA or IHCA, treated with ECPR in our 35-bed multidisciplinary ECPR from January 1, 2010, to January 31, 2024. Exclusion criteria were ECPR for hypothermic CA (*n* = 5 patients) and refusal to participate in any form of research, as specified in the patient’s medical file (*n* = 5 patients). The final cohort comprised a total of 166 patients (Fig. 1, flowchart). Before July 1st, 2017 (“Period 1”, *n* = 45 patients), the decision to implement ECPR was at the discretion of the physicians in charge. Starting July 1st, 2017 (“Period 2”, *n* = 121 patients), a clinical protocol was introduced to aid clinicians in their decision to initiate ECPR, but the final decision was left at the judgment of the medical team in charge of the patient. The protocol was based on the following 4 clinical criteria:

1. Age < 70 years
2. Shockable rhythm (VF/pulseless VT)
3. No flow < 5 min
4. Low-flow duration before initiation of VA-ECMO flow < 80 min.

Patients with unwitnessed cardiac arrest were not eligible for the ECPR pathway. No-flow time was defined as the interval between cardiac arrest onset and initiation of CPR by bystanders or professionals. In the absence of immediate CPR, the no-flow time was estimated as the time difference between the witnessed arrest and the beginning of CPR, and the information was entered in the patient’s file by healthcare providers. Regarding low flow time, it was defined as the time interval between the start of CPR and the achievement of full VA-ECMO flow.

The criteria were chosen from the available literature at the time. A witnessed arrest with no flow < 5 min was then considered mandatory to initiate ECPR [10]. The low-flow criterion (80 min) was based on studies reporting ECPR survival after periods exceeding 75 min [11, 12], as well as on some available protocols allowing up to 100 min low flow to start ECPR [12, 13]. The age criterion was selected upon existing ECPR protocols using a cutoff of 70 years [14–17]. With respect to initial rhythm, a shockable rhythm was known as a significant predictor of success of ECPR [18], although several authors had reported possible survival in patients with an initial PEA [12, 16, 19, 20]. Accordingly, a shockable rhythm was included as a major ECPR criterion in our protocol, but an initial PEA was not considered as an exclusion criterion (while asystole was) and clinicians could, therefore, include PEA patients based on their own judgment.

### VA-ECMO strategy

VA-ECMO was peripheral (femoro-femoral) in 162 patients and central in 4 patients. All cannulations were performed by a cardiac surgeon using a surgical cut-down technique. Systemic anticoagulation with intravenous heparin was given to achieve an Activated Clotting Time (ACT) of 180 or an anti-Xa activity of 0.3–0.5. Intravenous fluids and vasopressor agents (norepinephrine, epinephrine, vasopressin, alone or in combination) were given to maintain a mean blood pressure of 65 mm Hg, and analgo-sedation was maintained using Propofol (2–4 mg/kg/h) or Midazolam (0.05–0.15 mg/kg/h) and fentanyl (1.5–3.0 µg/kg/h). Mechanical ventilation was generally performed at a tidal volume of 6–8 ml/kg, a respiratory rate of 10–15/min and a positive end expiratory pressure (PEEP) of 6–10 cm H_2_O. Targeted temperature management after cardiac arrest evolved from 33 to 34 °C (until 2015) to 36 °C after 2015. ECMO was weaned when patients maintained a mean BP > 65 mm Hg and a left ventricle ejection fraction > 20% under minimal vasoactive support. After 2017, an additional criterion for ECMO weaning was an aortic velocity time integral (VTI) > 10 cm on transthoracic echocardiography.

### Data collection

Demographic variables (age and gender), location of CA (OHCA and IHCA), the cause and characteristics of CA (initial rhythm, duration of no flow, and low flow), causes of death, and the duration of ICU stay were recorded in each patient. The primary outcome was hospital survival. The secondary outcomes were day 90 survival and cerebral performance category (CPC score) at 3 months (CPC 1: full recovery or mild disability; CPC 2: moderate disability; CPC 3: severe disability; CPC 4: persistent vegetative state; CPC 5: death). Good neurological outcome was considered as a CPC score of 1 or 2.

### Data analysis

Continuous variables are expressed as medians and interquartile ranges (IQR), and categorical data as absolute numbers and percentages. Data were compared between period 1 and period 2 using the Wilcoxon’s rank sum test for continuous variables and the Pearson’s chi-square test for categorical variables. The presence or absence of each ECPR criteria (low flow < 80 min, shockable rhythm, no flow < 5 min, and age < 70 years) was determined in each patient in both period 1 and period 2. Hospital survival and 3 months CPC scores were analyzed according to each criteria present or absent (Pearson’s test). Furthermore, the proportions of surviving patients and patients with good neurological outcome (CPC 1 and 2) were compared according to the total number of criteria (0–4) present before the initiation of ECPR (Pearson’s test). Survival at day 90 was analyzed using Kaplan–Meier analysis and the log-rank test. Since PEA was not a formal exclusion criterion in our protocol, we performed additional analyses to specifically address survival and neurological outcomes in patients with an initial PEA who fulfilled the three other criteria of age, low flow, and no flow. Univariate and multivariable regression analyses were performed to determine the association of several explanatory variables with hospital survival as the dependent variable. Explanatory variables were included either as continuous variables (age, low flow, and no flow), or as dichotomous variables (no flow < 5 min; low flow < 80 min; age < 70 years). For each variable, odds ratios (OR) and 95% CI were determined, and the likelihood ratio test was used in the analyses. The JMP software, version 15, was used for all the analyses, and a p value < 0.05 was considered statistically significant.

## Results

Patient demographics, cardiac arrest characteristics, and outcomes are shown in Table 1 for the whole cohort (*n* = 166) and compared between period 1 (*n* = 45 patients) and period 2 (*n* = 121 patients). In the whole cohort, patients were predominantly male with a median age of 56 years, and IHCA (51.8%) was slightly more prevalent than OHCA (48.2%), while myocardial ischemia was the main cause of CA (53.0%). Whereas patients from both periods were comparable in terms of age, gender, ICU length of stay, and causes of CA, IHCA was significantly more prevalent in period 1 than period 2 (68.9 vs. 45.5%, *p* = 0.007), whereas the opposite was true for OHCA (31.1 vs. 54.5%, respectively, *p* = 0.007).

**Table 1:** Patient demographics, cardiac arrest characteristics, and outcomes

Variable		All pts (*n* = 166)	Period 1 (*n* = 45)	Period 2 (*n* = 121)	*p* value
Age (y), median (IQR)		56 (44–65)	55 (43–67)	56 (45–65)	0.886
Male gender, n (%)		127 (76.5)	35 (77.8)	92 (76.0)	0.814
OHCA, n (%)		80 (48.2)	14 (31.1)	66 (54.5)	0.007
IHCA, n (%)		86 (51.8)	31 (68.9)	55 (45.5)	0.697
CA etiology, *n* (%)					0.697
* Myocardial ischemia*		88 (53.0)	20 (44.4)	68 (56.2)	
* Hypoxia*		18 (10.8)	4 (8.9)	14 (11.6)	
* Pulmonary embolism*		15 (9.0)	5 (11.1)	10 (8.3)	
* Perioperative*		8 (4.8)	4 (8.9)	4 (3.3)	
* Intoxication*		5 (3)	1 (2.2)	4 (3.3)	
* Acute aortic syndrome*		4 (2.4)	1 (2.2)	3 (2.5)	
* Undetermined*		8 (4.8)	3 (6.7)	5 (4.1)	
Others*		20 (12.0)	7 (15.6)	13 (10.7)	
ICU LOS (d), median (IQR)		3.7 (0.9–17.8)	2.7 (0.6–10.8)	4.4 (1.2–18.2)	0.163
Survival, *n* (%)		36 (21.7)	4 (8.9)	32 (26.5)	0.015
Survival OHCA, *n* (%)		17 (21.3)	1 (7.1)	16 (24.2)	0.155
Survival IHCA, *n* (%)		19 (22.1)	3 (9.7)	16 (29.1)	0.037
CPC 1–2, *n* (%)		21 (12.7)	3 (6.7)	18 (14.9)	0.157
CPC 3, *n* (%)		16 (9.6)	1 (2.2)	15 (12.4)	0.048
CPC 4–5, *n* (%)		129 (77.7)	41 (91.1)	88 (72.7)	0.011
No flow (min), median (IQR)		0 (0–0)	0 (0–0)	0 (0–1)	0.034
Low flow (min), median (IQR)		60 (45–80)	60 (45–90)	60 (45–76)	0.498
No flow < 5 min, n (%)		150 (91.4)	43 (95.6)	107 (89.9)	0.249
Low flow < 80 min, n (%)		126 (75.9)	30 (66.7)	96 (79.3)	0.089
Age < 70 years		144 (86.7)	38 (84.4)	106 (87.6)	0.594
Shockable rhythm, *n* (%)		73 (44.5)	15 (34.1)	58 (48.3)	0.103
Non shockable rhythm, *n* (%)		91 (55.5)	29 (65.9)	62 (51.7)	0.103
PEA, *n* (%)		66 (40.2)	18 (40.9)	48 (40.0)	0.916
Asystole, *n* (%)		25 (15.2)	11 (25.0)	14 (11.7)	0.035

*CA* Cardiac Arrest, *CPC* Cerebral Performance Category, *ICU* Intensive Care Unit, *IHCA* In-Hospital Cardiac Arrest, *IQR* Interquartile Range, *LOS* Length of Stay, *min* minutes, *OHCA* Out-of-Hospital Cardiac Arrest, *PEA* Pulseless Electrical Activity, *y* years

^*^Other causes of CA: Anaphylactic shock, *n* = 2; aortic stenosis, *n* = 2; Central Nervous System pathology, *n* = 4; myocarditis, *n* = 1; primary arrhythmia, *n* = 2; Takotsubo cardiomyopathy, *n* = 1; cardiac allograft rejection, *n* = 2; hyperkalemia, *n* = 1; Left Ventricle Assist Device dysfunction, *n* = 1; obstructive cardiomyopathy, *n* = 1; thyroid storm, *n* = 1; septic shock, *n* = 2

**Fig. 2:**
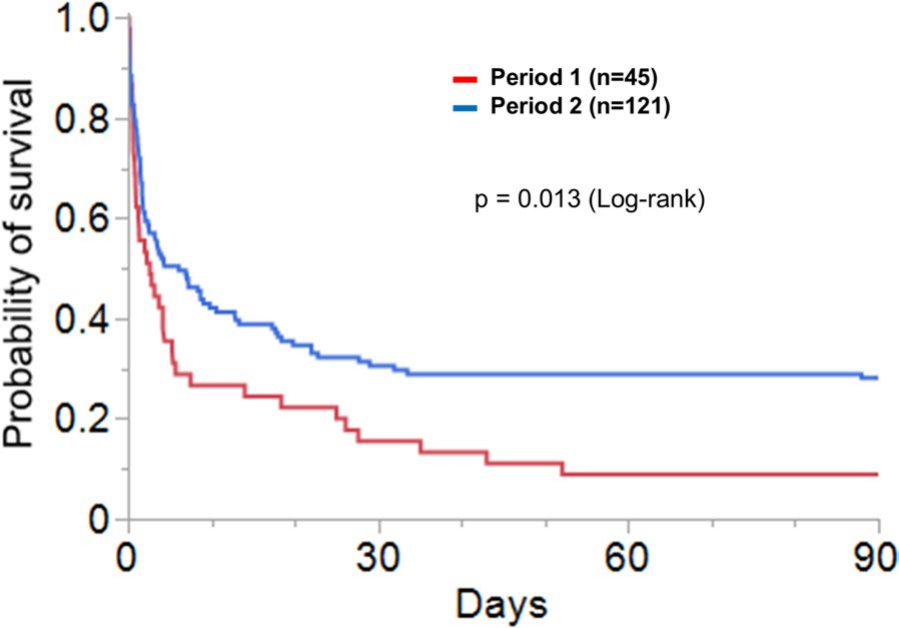
Kaplan–Meier plot of 90-day survival in periods 1 and 2

Overall, 36 patients (21.7%) survived to hospital discharge, comprising 17 OHCA survivors (21.3%) and 19 IHCA survivors (22.1%), with a good neurological outcome (CPC 1–2) in 21 patients (12.7%). Hospital survival was significantly higher in period 2 (26.5 vs. 8.9%, *p* = 0.015), which is further illustrated in Kaplan–Meier plot of day 90 survival during the two periods (Fig. 2). Survival improvement in period 2 was especially noted in patients with IHCA (29.1% survival in period 2 vs. 9.7% in period 1, *p* = 0.037), whereas survival for OHCA was not significantly different (24.2 vs. 7.1% in period 2 and 1, respectively, *p* = 0.155). CPC scores showed a non-significant increase in CPC 1–2 in period 2 (14.9 vs. 6.7%, *p* = 0.157), while there were significantly more patients with CPC 3 (12.4 vs. 2.2%, *p* = 0.048) and less patients with CPC 4–5 (72.7 vs. 91.1%, *p* = 0.011).

As indicated in Additional File 1, the causes of death included predominantly refractory circulatory shock, brain death and severe neurological injury (anoxic encephalopathy and massive stroke). In all patients, death was consecutive to withdrawal of life-sustaining therapies (WDLST) or withholding of life-sustaining therapies (WHLST), when therapies were deemed futile following multidisciplinary discussion and inclusion of family members whenever possible. In patients with recurrent refractory (in-hospital) CA, reestablishment of ECPR was withheld. In two patients developing septic shock and in one patient developing delayed respiratory failure, specific therapies were withheld owing to the patients’ very poor overall condition. In patients with refractory circulatory shock, severe hemorrhagic shock, septic shock, and one case of ECMO malfunction, WDLST was decided upon the overall clinical context and the impossibility to maintain arterial blood pressure despite maximal vasopressor support. For the specific group of patients with anoxic encephalopathy, WDLST relied on multimodal neuroprognostication, when patients presented at least two of the following prognostic markers of poor neurological outcome [21]: absent pupillary and corneal reflexes, bilaterally absent somatosensory evoked potentials, malignant electroencephalography patterns, diffuse and extensive anoxic injury on brain CT scan, or Magnetic Resonance and high peak serum neuron-specific enolase (> 60 μg/L). In patients developing a massive stroke (ischemic or hemorrhagic), WDLST was decided upon the general clinical context and the severity of the neurological injury. In 21 patients (19.2% of non-survivors), death occurred after ECMO weaning. Organ donation was obtained from 4 non-surviving patients (3.1%).

**Fig. 3:**
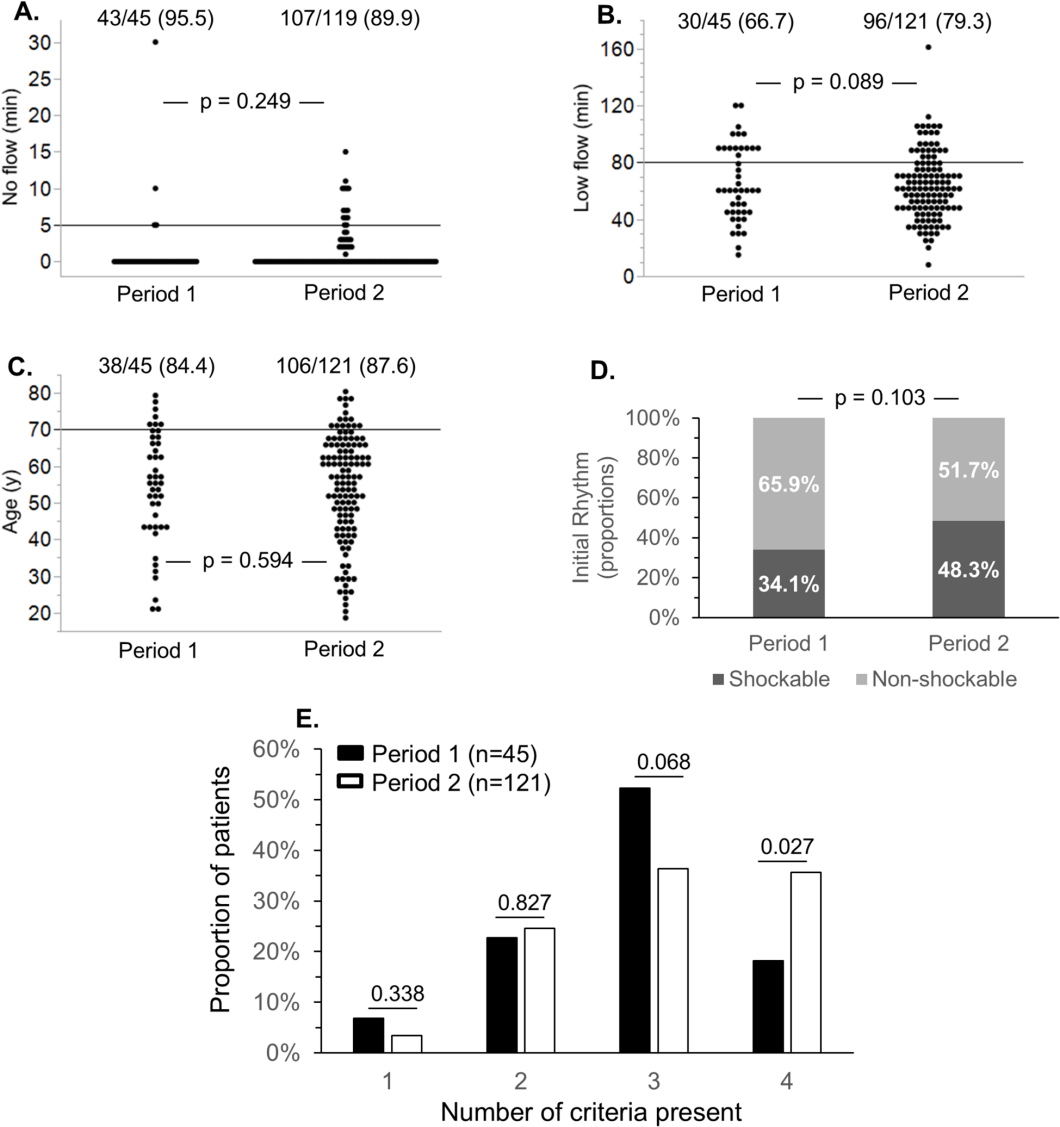
Fulfillment of ECPR criteria before and after protocol implementation. Individual data points for no flow (**A**), low flow (**B**), and age (**C**). The horizontal lines indicate the cut-off values for each recommended criterion (no flow: 5 min; low flow: 80 min; age: 70 years). The proportions of patients with a shockable or a non-shockable rhythm are shown in (**D**), and the proportion of patients fulfilling 1, 2, 3, or 4 criteria is shown in (**E**). *P* values indicate comparisons of the proportions of patients in period 1 and 2 with each indicated criterion. The four criteria were: a no flow < 5 min, a low flow < 80 min, an age < 70 years, and a shockable rhythm. The number of criteria (1–4) refers to the presence of 1–4 of any of these criteria. No flow was missing in 2 patients from period 2. Initial rhythm was missing in 1 patient from period 1 and 1 patient from period 2. *min* minutes, *y* years

To assess the adherence to our recommended ECPR criteria in period 2, we compared the proportions of patients fulfilling each individual criteria in both periods. As indicated in Table 1 and Fig. 3A–D, the proportion of patients with a no flow < 5 min (Fig. 3A), a low flow < 80 min (Fig. 3B), an age < 70 years (Fig. 3C), and a shockable rhythm (Fig. 3D) were not statistically different between the two periods. Regarding initial rhythm, the only significant difference was a lower proportion of patients with asystole in period 2 compared to period 1 (11.7 vs. 25.0%, *p* = 0.035, Table 1). While individual criteria were comparably present in both periods, Fig. 3E shows that the simultaneous presence of 4 criteria was significantly more prevalent in period 2 than in period 1 (35.0 vs. 17.8%, *p* = 0.027), while the presence of only 3 criteria tended to be more prevalent in period 1 (*p* = 0.068). Of note, 2 patients in period 2 had missing data for no-flow duration and one patient from period 1 had missing data on initial rhythm, but these 3 patients did not fulfill at least one of the other 3 criteria. These 3 patients were, therefore, categorized as < 4 criteria. One patient from period 2 had missing data regarding initial rhythm but fulfilled the other 3 criteria. This patient could therefore not be categorized as 4 or < 4 criteria.

**Fig. 4:**
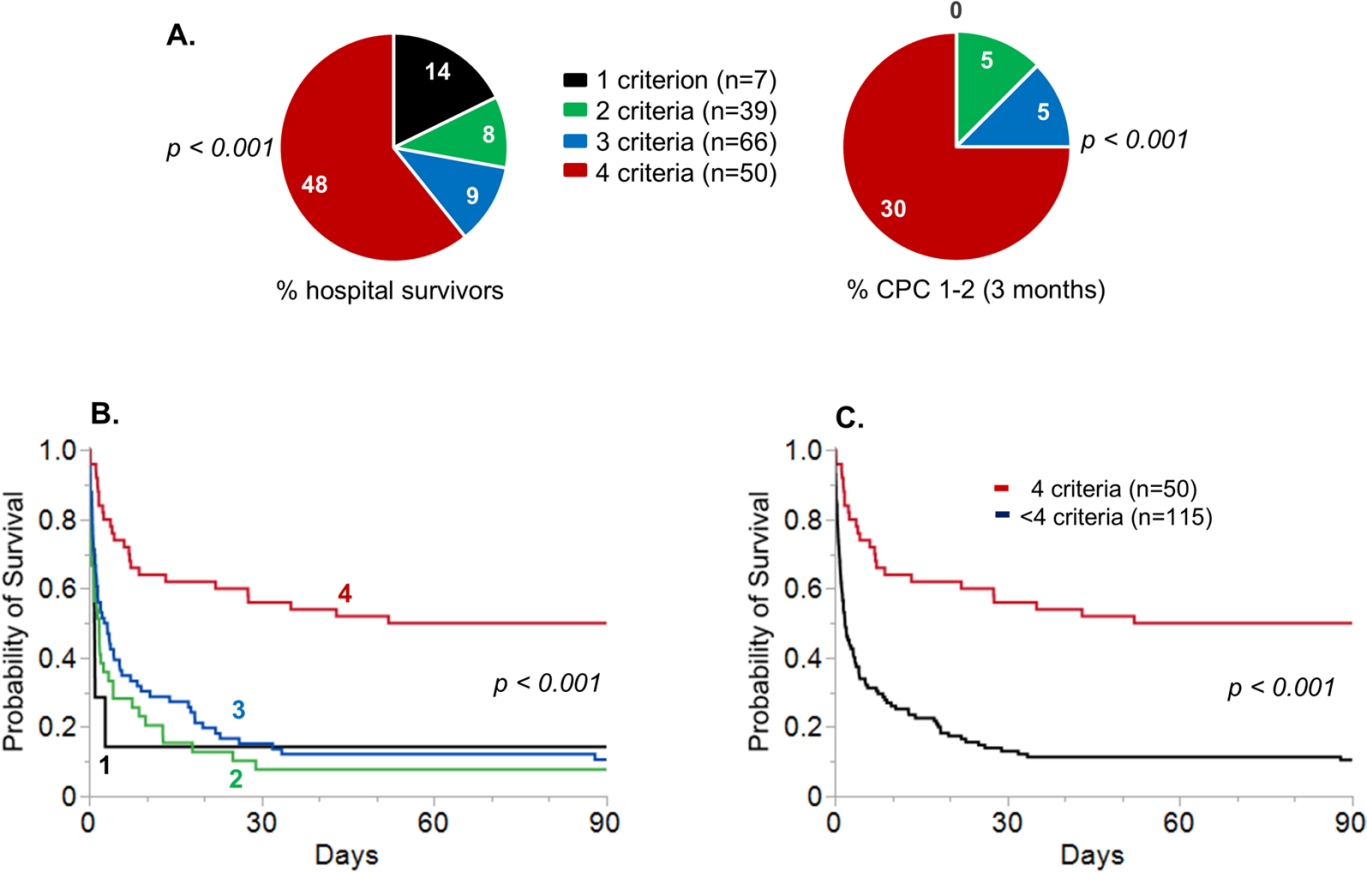
Hospital survival and good neurological outcome according to the number of ECPR criteria during the whole study period. **A** Proportion of hospital survivors and of patients with good neurological outcome (**A**), according to the number of ECPR criteria (age < 70 years, no flow < 5 min, low flow < 80 min, and shockable rhythm). **B** Kaplan–Meier plot of day 90 survival according to the number of ECPR criteria. **C** Kaplan–Meier plot of day 90 survival according to the presence or absence of the 4 ECPR criteria. In 4 patients (2 in each period), the total number of criteria could not be computed due to missing data (2 initial rhythms and 2 no flows), but 3 of them did not fulfill at least one other criterion and were, therefore, categorized as < 4 criteria. One patient fulfilled the 3 other criteria and could not be classified as 4 or < 4 criteria. *CPC *Cerebral Performance Category, *ECPR* Extracorporeal Cardio-Pulmonary Resuscitation, *min* minutes, *y* years

The proportions of patients surviving to hospital discharge and patients with a CPC 1–2 (good neurological outcome) according to the number of ECPR criteria during the two periods (collectively analyzed) are shown in Fig. 4A. While patients fulfilling only 1, 2, or 3 criteria had very low survival and good neurological outcome, patients fulfilling the 4 criteria had a 48% hospital survival, with a good neurological outcome of 30%, a highly significant difference compared to 1, 2, or 3 criteria (*p* < 0.001). Kaplan–Meier survival curves (Fig. 4B, C) further illustrate the marked improvement of day 90 survival in patients fulfilling the 4 criteria when compared to only 1, 2, or 3 criteria (Fig. 4B, C), or when comparing the presence of 4 versus < 4 criteria (Fig. 4D, E).

The clinical and cardiac arrest characteristics, as well as outcomes of patients with IHCA and OHCA are shown in Additional File 2. Patients with IHCA were significantly older, displayed shorter no-flow and low-flow times, and presented significantly more often with a non-shockable rhythm. Despite these differences, hospital survival and good neurological outcome were comparable in the two populations of patients. Furthermore, the proportion of patients fulfilling our 4 ECPR criteria was not significantly different between IHCA and OHCA, and these patients displayed comparable survival and neurological outcome.

**Fig. 5:**
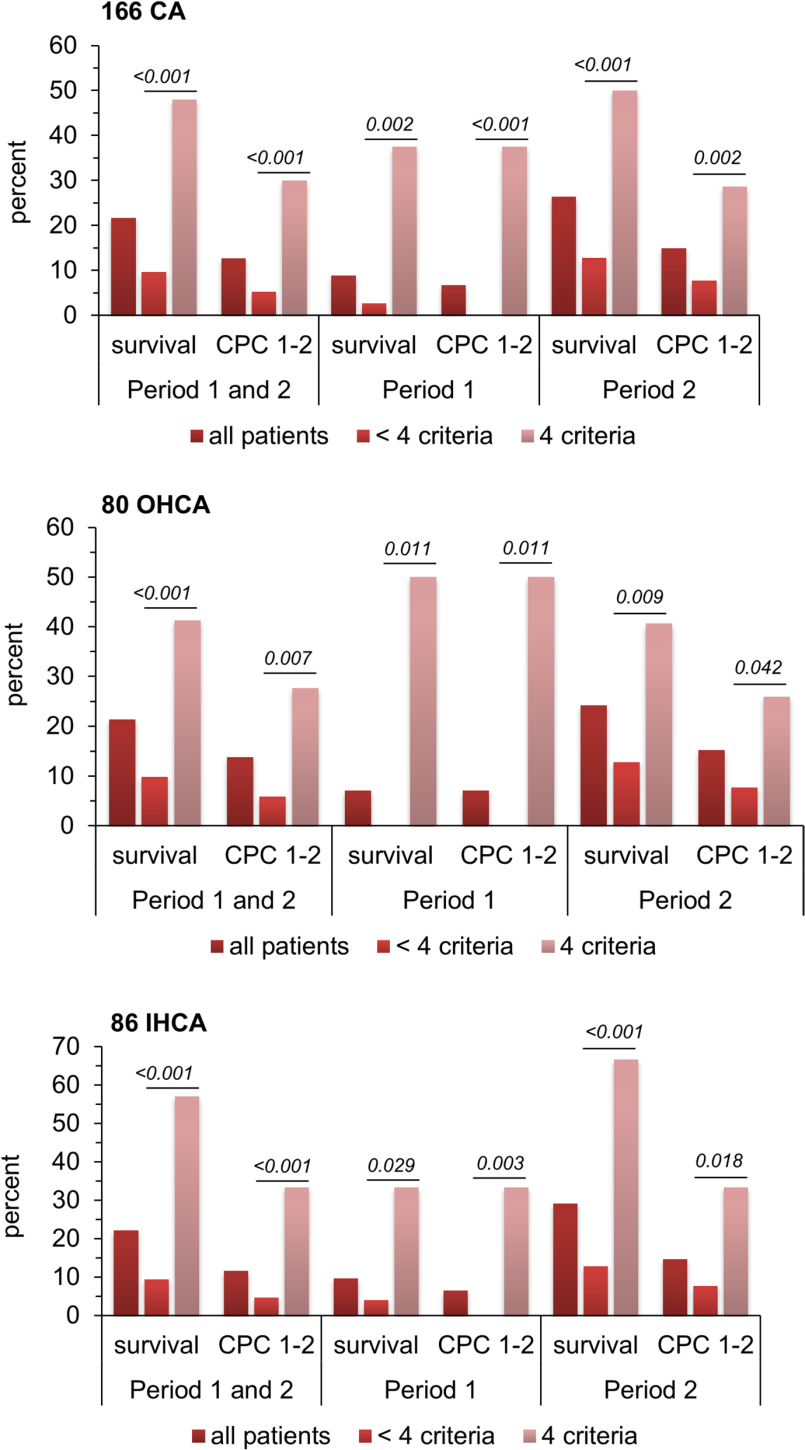
Proportion of survivors and patients with good neurological outcome (CPC 1–2), according to period, type of CA, and the presence or absence of the 4 ECPR criteria. **A** Whole population of Cardiac Arrest (CA). **B** Patients with Out-of-Hospital Cardiac Arrest (OHCA). **C** Patients with In-Hospital Cardiac Arrest (IHCA). The columns indicate the proportions of patients in each category, and the number above the columns indicate the numbers of survivors and patients with Cerebral Performance Category (CPC) of 1–2 over the total number of patients in each category. 1 patient with IHCA from period 2 fulfilled 3 criteria but had missing data for the 4th criterion and could, therefore, not be categorized as 4 or < 4 criteria

Figure 5 illustrates the influence on survival and good neurological outcome of the presence of 4 criteria in period 1 and period 2 in the whole population of CA (Fig. 5A), and in the population of patients with OHCA (Fig. 5B) and IHCA (Fig. 5C). Irrespective of the period and the type of CA (OHCA or IHCA), the presence of 4 criteria before the implementation of ECPR was associated with highly significant improvements in both survival and good neurological outcome.

**Fig. 6:**
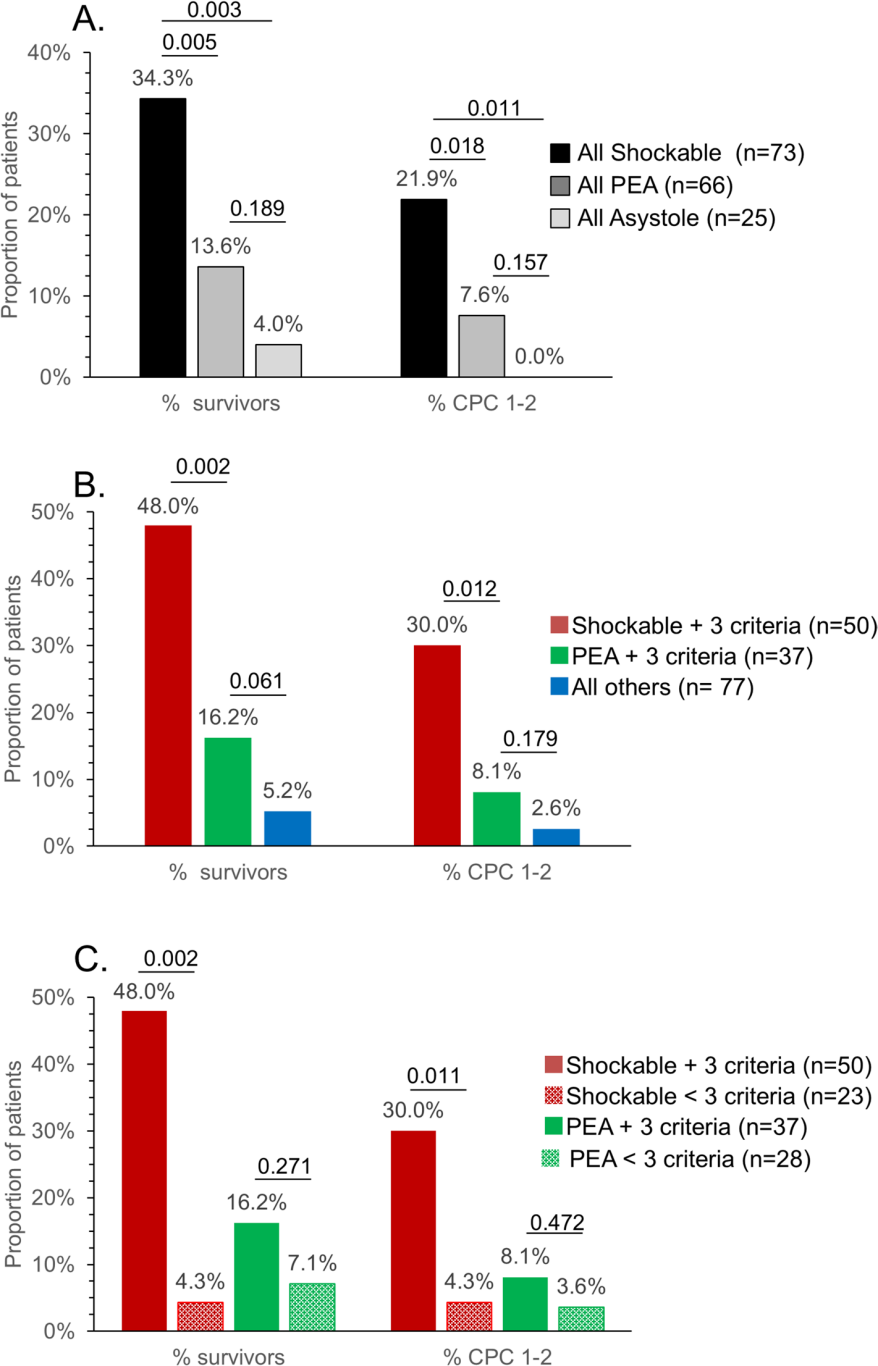
Outcomes of ECPR in patients with pulseless electrical activity as the initial rhythm. **A** Hospital survival and good neurological outcome (CPC 1–2) in patients with shockable rhythm, PEA, or asystole. **B** Hospital survival and neurological outcome in patients fulfilling 4 ECPR criteria (shockable rhythm, age < 70 years, no flow < 5 min, and low flow < 80 min), in patients with PEA fulfilling the 3 other criteria (no flow < 5 min, low flow < 80 min, and age < 70 years) and in all the other patients with 3 or < 3 criteria. **C** Influence of fulfilling the 3 criteria of age (< 70 years), no flow (< 5 min), and low flow (< 80 min) in patients with shockable rhythm or PEA. Values above the columns indicate the proportions in each category of patients. Two patients had missing data for rhythm and could not be included in (**A**). One PEA patient with a missing data for no flow could not be categorized in (**B**, **C**). *CPC* Cerebral Performance Category, *ECPR* Extracorporeal Cardio-Pulmonary Resuscitation, *PEA* Pulseless Electrical Activity

Analyses of outcomes in PEA patients are shown in Fig. 6. We first assessed hospital survival and CPC 1–2 according to initial rhythm (Fig. 6A). Patients with a shockable rhythm had significantly better survival and neurological outcome than those with a non-shockable rhythm, and among the latter group, patients with PEA did not significantly differ from those with asystole. We then compared outcomes of PEA patients fulfilling the 3 other criteria (age, no-flow, and low-flow) to patients with the 4 recommended criteria and to those with 3 or < 3 criteria (Fig. 6B). Patients with PEA and the 3 other criteria had significantly reduced survival and neurological outcome in comparison to patients with 4 criteria. In comparison to all other patients, they showed a trend for better hospital survival (16.2 vs. 5.2%, *p* = 0.061), but no significant improvement in good neurological outcome (8.2 vs. 2.6%, *p* = 0.179). Finally, we compared the impact of the 3 criteria of age, no flow, and low flow in patients with either a shockable rhythm or a PEA (Fig. 6C). Whereas patients with a shockable rhythm had significant increases in survival and CPC 1–2 if they fulfilled the 3 other criteria, the outcomes were statistically comparable in PEA patients regardless of the presence or absence of the three other criteria.

The results of univariate and multivariable analyses are shown in Table 2. In univariate analyses (Table 2A), age and low-flow duration were significantly associated with mortality, whereas a shockable rhythm and treatment during period 2 presented a significant association with a decreased risk of mortality. By contrast, no significant associations with hospital mortality were found for gender, CA location (IHCA vs OHCA), and no-flow duration (which was shorter than 5 min in most patients). Multivariable adjustment analysis (Table 2A) showed that each additional year of age was associated with a 5% increase in the odds of death, and each additional minute of low flow increased the odds of death by 5%. A shockable rhythm was significantly associated with a decreased mortality risk (OR = 0.14, 95% CI 0.05–0.35, *p* < 0.001). For the same age, low-flow time, and type of rhythm, treatment during period 2 was clearly associated with better outcomes, showing a significant decrease in mortality (OR = 0.28, 95% CI 0.07–0.87, *p* = 0.027).

**Table 2:** Variables associated with in-hospital mortality: univariate and multivariable analyses

	Univariate			Multivariable		
Variable	OR	95% CI	*p* value	OR	95% CI	*p* value
A.						
Age (per years)	1.03	[1.00–1.05]	0.039	1.05	[1.02–1.09]	0.002
Male gender	0.59	[0.21–1.45]	0.261			
OHCA versus IHCA	1.05	[0.50–2.22]	0.895			
No flow (per min)	1.03	[0.92–1.20]	0.645			
Low flow (per min)	1.03	[1.01–1.05]	0.001	1.05	[1.02–1.07]	< 0.001
Shockable rhythm	0.24	[0.10–0.52]	< 0.001	0.14	[0.05–0.35]	< 0.001
Period 2 versus period 1	0.27	[0.08–0.74]	0.009	0.28	[0.07–0.87]	0.027
B. Analysis according to protocol criteria						
Shockable rhythm	0.24	[0.10–0.52]	< 0.001	1.13	[0.16–9.57]	0.900
Age < 70 years	0.32	[0.05–1.19]	0.095	1.06	[0.15–5.13]	0.948
No flow < 5 min	0.59	[0.09–2.31]	0.482	2.77	[0.34–17.18]	0.311
Low flow < 80 min	0.14	[0.02–0.50]	0.001	0.37	[0.04–2.01]	0.267
Presence of 4 criteria	0.11	[0.05–0.26]	< 0.001	0.11	[0.01–0.87]	0.037

*CI* Confidence Interval, *ECPR* Extracorporeal Cardio-pulmonary Resuscitation, *IHCA* In-Hospital Cardiac Arrest, *min* minutes, *OHCA* Out-of-Hospital Cardiac Arrest, *OR* Odds Ratio, *y* years, *min* minutes

A. The multivariable model was adjusted for age (years) and the duration of low flow (minutes) (both as continuous variables), shockable rhythm and study period (period 2 vs. period 1) (both as dichotomous variables). Hospital survival was the dependent variable

B. The multivariable model was adjusted for shockable rhythm, age < 70 years, no flow < 5 min, low flow < 80 min, and the presence of the four protocol criteria (all as dichotomous variables). Hospital survival was the dependent variable

Table 2B shows the results of the impact of each ECPR criterion separately, and those of the simultaneous presence of all four criteria. In univariate analysis, a significantly lower risk of mortality was associated with a shockable rhythm, low flow < 80 min, and with the presence of four criteria, while no significant association was found for an age < 70 years and a no flow < 5 min. In the multivariable analysis, only the simultaneous presence of the four criteria was significantly associated with a decreased risk of death (OR = 0.11, CI 0.01–0.87, *p* = 0.037), with no significant association found for each criterion separately, which highlights the critical importance of fulfilling the 4 criteria to positively impact hospital survival after ECPR.

Since we chose a low flow < 80 min as an ECPR criterion, we examined whether a shorter low flow (< 60 min) would have provided a greater benefit. We, therefore, compared patients fulfilling the no flow, rhythm, and age criteria together with a low flow < 60 min or a low flow comprised between 60 and 80 min. As shown in Additional File 3, patients with a low flow < 60 min had better survival and good neurological outcome than those with a low flow of 60–80 min, but the difference did not reach statistical significance (survival, *p* = 0.068; CPC 1–2, *p* = 0.279). Furthermore, we also estimated the adjusted probabilities of hospital survival using a logistic regression model including an interaction between the low-flow duration as a continuous variable and the simultaneous presence or absence of the three other ECPR criteria as binary variables (age > or < 70 years, shockable or non-shockable rhythm, and no flow > or < 5 min). Predicted values were generated using the margin command for low-flow durations ranging from 0 to 100 min (10-min increments). As shown in Additional File 4, patients not fulfilling these three ECPR criteria exhibited a lower predicted survival as low-flow duration increased. Moreover, for low-flow durations up to approximately 70 min, the presence or absence of these three criteria discriminated survival probability substantially; however, this discriminatory effect progressively diminished and disappeared as low-flow duration became longer.

## Discussion

The main results of this before-and-after, single-center observational analysis indicate that a structured ECPR protocol comprising four simple criteria based on initial rhythm, age, and durations of no flow and low flow significantly improved survival in patients with refractory cardiac arrest. The proportion of patients fulfilling these 4 ECPR criteria was significantly increased after the implementation of the protocol, although adherence to the protocol remained relatively low (35.0%, contrasting with 17.8% before protocol implementation). Patients who met the 4 defined criteria (both before and after protocol) had significantly better hospital survival and 3 months neurological outcomes, irrespectively of the location of cardiac arrest (IHCA or OHCA). These results are not intended to define universal ECPR selection criteria, but they indicate associations between a locally developed protocol and ECPR outcomes in one single institutional practice.

Evidence regarding the impact of ECPR on survival and neurological outcome in patients with refractory CA remains limited. Three recent randomized-controlled trials (RCTs) comparing ECPR to conventional CPR in OHCA [7–9] yielded contrasted results [22], while observational studies are the only resources available for ECPR in IHCA [23, 24]. As a result, current recommendations to select patients for ECPR remain based on this low level of evidence [25–28]. Generally accepted patient selection criteria include an age < 65 years, witnessed arrest with no or very brief no-flow time, a low-flow time < 60 min, shockable rhythm, and the absence of severe acute or chronic co-morbidities [29]. Several groups have published their local protocol for patient selection and contraindication for ECPR, showing a major impact on patient outcome. Assouline et al. [30] compared a permissive (no flow ≤ 3 min, low flow ≤ 100 min, and age ≤ 65 years) to a restrictive (no flow 0 min, low flow ≤ 60 min, and age ≤ 55 years) ECPR selection algorithm in 48 OHCA patients and showed a major impact on 30-day survival with the restrictive approach (68% vs. 9%). In a study on 35 IHCA/OHCA patients, Akhtar et al. reported that applying rigorous exclusion criteria (asystole as the initial rhythm, no bystander CPR, and low-flow time > 60 min) increased survival of ECPR from 9.1 to 69.2% [31].

Our study further emphasizes the key role of a dedicated ECPR protocol to select patients at higher chance of meaningful survival. Patients’ outcomes were improved only if the 4 protocol criteria were respected, but the benefit was cancelled if only one criterion was missing. This is important information, since clinicians frequently deviated from the protocol with the belief that ECPR could also benefit patients not fulfilling all selection criteria. Only 35% of patients were properly selected according to our protocol, which underscores the challenges to ensure physician adhesion in emergency situations.

The main deviation from the protocol was that an initial non-shockable rhythm was present in more than half of our population (51.7%). Asystole was a definitive exclusion criterion in our protocol, but 11.7% of patients (14/121) still underwent ECPR despite initial asystole. None of them showed a good outcome, substantiating that these patients should not be candidates for ECPR, in agreement with current recommendations [29]. Regarding PEA, literature at the time of protocol enforcement suggested that some PEA patients might benefit from ECPR [12, 16, 19, 20]. Therefore, PEA was not mentioned as definitively exclusive for ECPR and inclusion of such patients was left at the judgment of the treating physicians. This somewhat unclear feature of our protocol resulted in a high proportion of PEA patients undergoing ECPR after protocol enforcement (40%), not different from the proportion before protocol (40.9%).

We found that patients with an initial PEA had a dismal prognosis, with a good neurological outcome occurring only in 7.6%. The negative impact of PEA persisted regardless of the presence of the three other criteria (age, no flow, and low flow), in striking contrast with the major benefit in patients with a shockable rhythm who fulfilled these three criteria. While these findings do not support the widespread use of ECPR in PEA patients, a small minority (5/66) still benefited from this intervention. Therefore, PEA might not be a definitive exclusion criterion for ECPR, but factors distinct from age, no flow, and low flow should be determined to select PEA candidates for ECPR. In this prospect, Kawauchi et al. recently proposed a straightforward 3-factor score (START-ECPR score) to predict a favorable outcome of ECPR in PEA patients [32]. The score included signs of life at hospital arrival, absence of asystole, and transient episodes of ROSC (> 1 min) during CPR. Patients with a score of 2 or 3 points had a 30.8% hospital survival and a 19.5% favorable neurological outcome.

Our protocol allows a total low-flow time of 80 min, above the 60 min limit recommended by many expert centers [29]. When comparing patients fulfilling the criteria for age, no flow and shockable rhythm, those with a low flow < 60 min had a better outcome than those with a low flow between 60 and 80 min, but the differences were not significant (survival 58.1 vs. 31.6%, *p* = 0.069; good neurological outcome 35.5 vs. 21.1%, *p* = 0.279). Using a cutoff for low flow of 60 min would have, therefore, excluded patients eventually surviving with a good neurological outcome. This assertion is reinforced by our analysis of the predicted probability of hospital survival according to low-flow duration, stratified by the presence or absence of the three other criteria of age, shockable rhythm, and no-flow. In the presence of these three criteria, survival probability was still increased in patients with low-flow times above 60 min. Therefore, we suggest that in patients < 70 years presenting with a shockable rhythm and a no flow of less than 5 min, a low flow up to 80 min might not be considered as a definitive exclusion criterion for ECPR.

We did not notice significant differences between ECPR outcomes of patients with IHCA and OHCA. In both cohorts, patients fulfilling our four defined criteria had comparable survival (IHCA, 57.1%; OHCA, 41.4%; *p* = 0.271) and good neurological outcomes (IHCA, 33.3%; OHCA, 27.6%; *p* = 0.662). While patients with IHCA had significantly shorter low-flow times than those with OHCA, in line with the previous findings [33], this did not translate into better ECPR outcomes, possibly due to the older age and more frequent non-shockable rhythms in IHCA patients, as reported by others [34]. In addition, IHCA patients generally present additional co-morbidities and complications that may jeopardize the success of ECPR, as outlined by Shi et al. in a large observational study comparing the outcomes of ECPR in IHCA and OHCA [35]. Owing to such differences in the epidemiology and cardiac arrest characteristics, decision to implement ECPR might require distinct selection criteria for OHCA and IHCA patients [33], an issue that should be addressed in the future.

Limitations to our study include, first, its retrospective nature and limited sample size. Second, due to the extended period of observation (14 years), it is possible that overall improvements in CPR and ECMO management may have influenced the outcome in the most recent years. Third, we did not assess functional outcomes beyond 3 months. Since up to 30% of cardiac arrest survivors have a change in their outcome between 1 and 6 months [36], we cannot exclude that some ECPR survivors in our study may have evolved toward a different functional outcome over the long term. Finally, despite the implementation of protocolized selection criteria, a significant proportion of patients received ECPR outside these criteria. One reason was the lack of formal exclusion of PEA patients, which resulted in a high proportion of such patients undergoing ECPR in our study. This highlights the persistent challenge of clinical judgment in real-world decision-making and underscores the ethical and practical complexities of rigid protocol adherence versus individual physician choices and decisions.

In conclusion, the implementation of a structured ECPR protocol was associated with improved ECPR survival rates, particularly among patients meeting predefined selection criteria. The combined effect of these criteria appears to be a key determinant of survival, reinforcing the necessity of systematic patient selection. However, ethical and logistical challenges persist, emphasizing the need for ongoing evaluation of ECPR protocols to ensure both efficacy and equity in patient care. Future research should focus on refining patient selection models to align clinical efficacy with ethical considerations.

## Supplementary Information

Additional file1 (DOCX 15 kb)

Additional file2 (DOCX 17 kb)

Additional file3 (TIF 451 kb)

Additional file4 (TIF 4146 kb)

## Data Availability

All data generated or analyzed during this study are included in this published article and its additional information files.

## Abbreviations

CA: Cardiac Arrest
CI: Confidence interval
CPR: Cardio-pulmonary Resuscitation
CPC: Cerebral Performance Category
ECPR: Extracorporeal Cardio-pulmonary Resuscitation
ICU: Intensive Care Unit
IHCA: In-Hospital Cardiac Arrest
IQR: Interquartile Range
OHCA: Out-of-Hospital Cardiac Arrest
OR: Odds Ratio
PEA: Pulseless Electrical Activity
RCT: Randomized-Controlled Trial
ROSC: Return Of Spontaneous Circulation
VA-ECMO: Veno-arterial Extracorporeal Membrane Oxygenation
VF: Ventricular Fibrillation
VT: Ventricular Tachycardia
