# Supplementary material for: Impact of a clinical decision protocol on survival and neurological outcome following extracorporeal cardiopulmonary resuscitation
Source: J Intensive Care. 2026 Mar 16;14:38. doi: 10.1186/s40560-026-00874-7 (PMC13097924; doi:10.1186/s40560-026-00874-7)
Supplement: Supplementary file 1 — Additional file1 (DOCX 15 kb) [file 40560_2026_874_MOESM1_ESM.docx]

Additional File 1. Causes and processes of death, death after ECMO weaning and organ donation

Cause of death n (%) WDLST WHLST Organ donation

Refractory circulatory shock 54 (41.5) 54

Severe anoxic encephalopathy 27 (20.8) 27 1 (DCD)

Brain death 15 (11.5) 15 3 (DBD)

Stroke 10 (7.7) 10

Hemorrhagic shock 6 (4.6) 6

Septic shock 6 (4.6) 4 2

Refractory CA after ECMO weaning 6 (4.6) 6

ECMO malfunction 1 (0.8) 1

Mesenteric ischemia 1 (0.8) 1

Severe respiratory failure 1 (0.8) 1

Multiple organ failure 2 (1.5)

Refractory SE (metabolic cause) 1 (0.8) 1

Death after ECMO weaning, n (%) 25 (19.2)

Abbreviations: CA, Cardiac Arrest; ECMO: Extra-Corporeal Membrane Oxygenation; SE Status Epilepticus; DCD: Donation after Circulatory Death; DBD: Donation after Brain Death; WDLST, Withdrawal of Life-Sustaining Therapies; WHLST: Withholding of Life-Sustaining Therapies.
