# Supplementary material for: Impact of a clinical decision protocol on survival and neurological outcome following extracorporeal cardiopulmonary resuscitation
Source: J Intensive Care. 2026 Mar 16;14:38. doi: 10.1186/s40560-026-00874-7 (PMC13097924; doi:10.1186/s40560-026-00874-7)
Supplement: Supplementary file 2 — Additional file2 (DOCX 17 kb) [file 40560_2026_874_MOESM2_ESM.docx]

Supplementary Table 2. Clinical characteristics and outcomes in patients with IHCA and OHCA

| Variable | All pts (n=166) | OHCA (n=80) | IHCA (n=86) | p value |  |
| --- | --- | --- | --- | --- | --- |
| Age (y), median (IQR) | 56 (44-65) | 52 (41-62) | 60 (49-68) | 0.003 |  |
| Male gender, n (%) | 127 (76.5) | 61 (76.3) | 66 (76.7) | 0.940 |  |
| No flow (min), median (IQR) | 0 (0-0) | 0 (0-3) | 0 (0-0) | < 0.001 |  |
| Low flow (min), median (IQR) | 60 (45-80) | 69 (55-90) | 53 (39-70) | < 0.001 |  |
| No flow < 5 min, n (%) | 150 (91.4) | 64 (82.0) | 86 (100) | < 0.001 |  |
| Low flow < 80 min, n (%) | 126 (75.9) | 52 (65.0) | 74 (86.1) | 0.002 |  |
| Age < 70 y | 144 (86.7) | 77 (96.2) | 67 (77.9) | < 0.001 |  |
| Shockable rhythm, n (%) | 73 (44.5) | 46 (57.5) | 27 (32.1) | 0.001 |  |
| Non shockable rhythm, n (%) | 91 (55.5) | 34 (42.5) | 57 (67.9) | 0.001 |  |
|  | PEA, n (%) | 66 (40.2) | 24 (30.0) | 42 (50.1) | 0.001 |
|  | Asystole, n (%) | 25 (15.2) | 10 (12.5) | 15 (17.9) | 0.035 |
| Hospital survival | 36 (21.7) | 17 (21.3) | 19 (22.1) | 0.895 |  |
| CPC 1-2 | 21 (12.7) | 11 (13.8) | 10 (11.6) | 0.681 |  |
| 4 criteria | 50 (30.3) | 29 (36.3) | 21 (24.7) | 0.107 |  |
| Hospital survival with 4 criteria | 24 (48.0) | 12 (41.4) | 12 (57.1) | 0.271 |  |
| CPC 1-2 with 4 criteria | 15 (30.0) | 8 (27.6) | 7 (33.3) | 0.662 |  |

Abbreviations. CPC: Cerebral Performance Category; IHCA: In-Hospital Cardiac Arrest; IQR: Interquartile Range; min: minutes; OHCA: Out-Of-Hospital Cardiac Arrest; PEA: Pulseless Electrical Activity; pts: patients; y: years
