# Supplementary figures and images for: Impact of a clinical decision protocol on survival and neurological outcome following extracorporeal cardiopulmonary resuscitation
Source: J Intensive Care. 2026 Mar 16;14:38. doi: 10.1186/s40560-026-00874-7 (PMC13097924; doi:10.1186/s40560-026-00874-7)

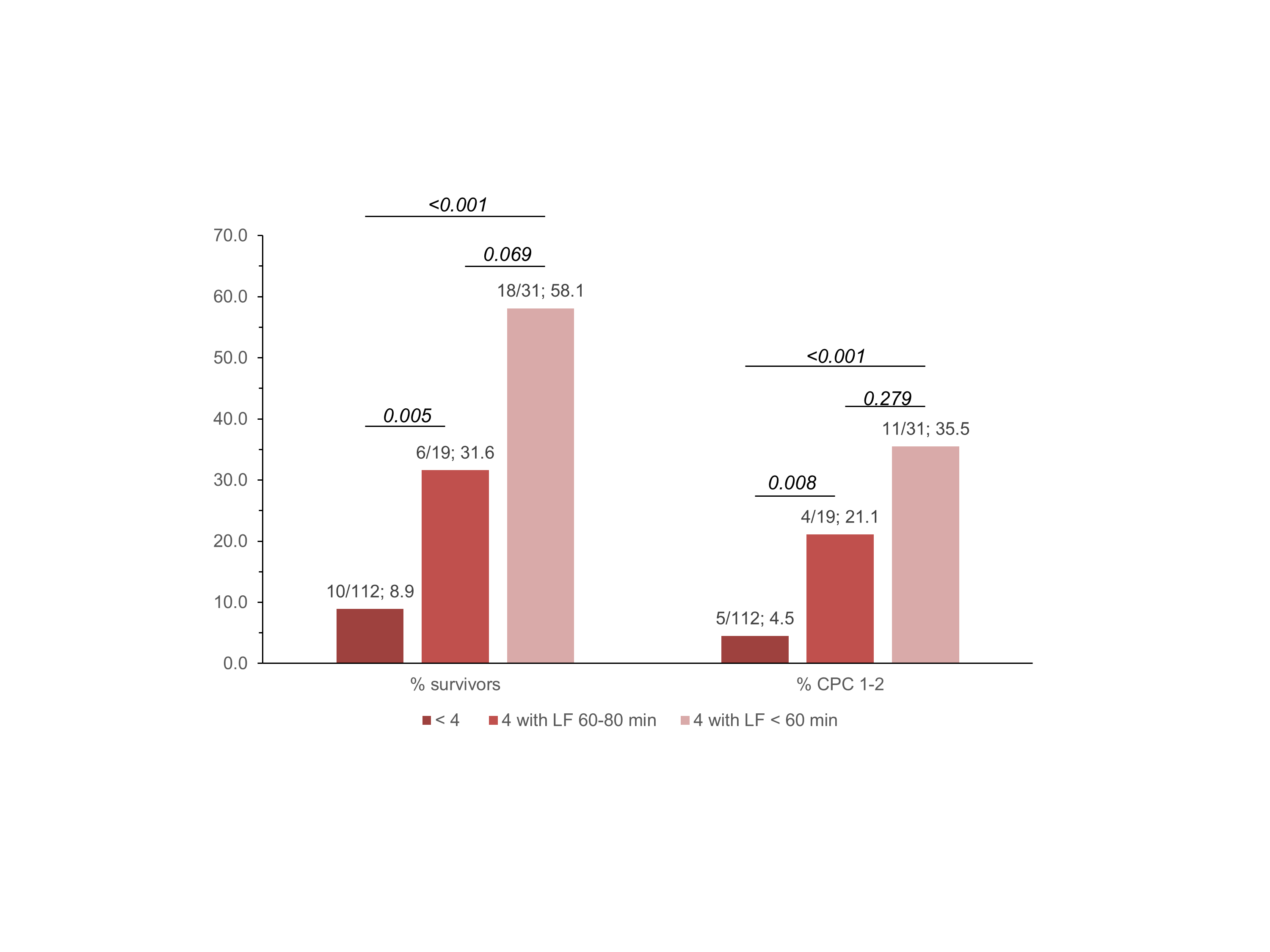

Supplement: Supplementary file 3 — Additional file3 (TIF 451 kb) [file 40560_2026_874_MOESM3_ESM.tif]

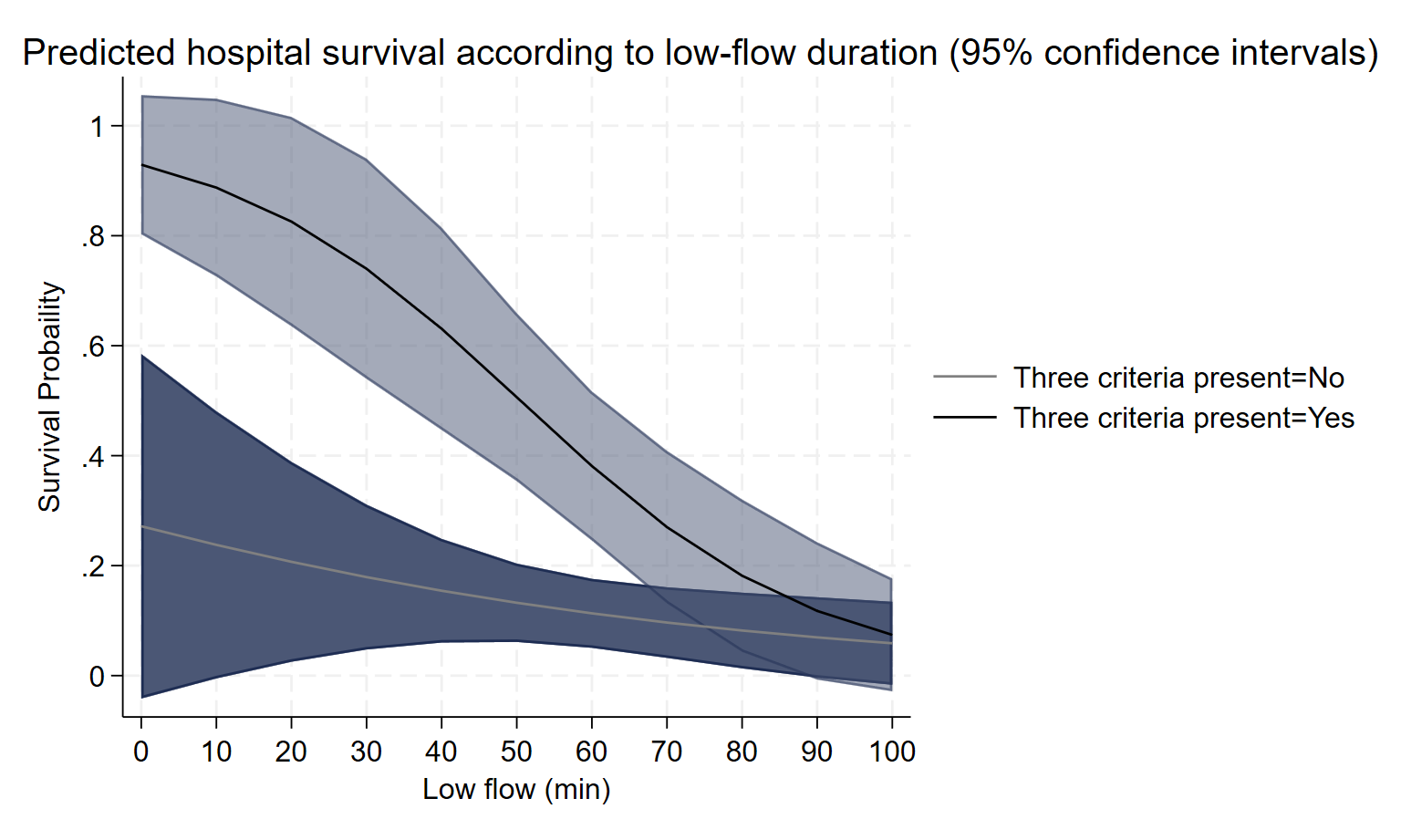

Supplement: Supplementary file 4 — Additional file4 (TIF 4146 kb) [file 40560_2026_874_MOESM4_ESM.tif]
